# Understanding Long-Term Survival in ALS: A Cohort Study on Subject Characteristics and Prognostic Factors

**DOI:** 10.3390/jcm14207351

**Published:** 2025-10-17

**Authors:** Elisabetta Pupillo, Elisa Bianchi, Maurizio Angelo Leone, Massimo Corbo, Massimiliano Filosto, Alessandro Padovani, Barbara Risi, Marcella Vedovello, Valentina dell’Era, Federica Cerri, Claudia Morelli, Luca Diamanti, Mauro Ceroni, Yuri Falzone, Andrea Rigamonti, Eugenio Vitelli

**Affiliations:** 1 Research Center for ALS, Laboratory of Neurological Disorders, Istituto di Ricerche Farmacologiche Mario Negri IRCCS, Via Mario Negri 2, 20156 Milan, Italy; 2 Need Institute, Foundation for the Cure and Rehabilitation of Neurological Diseases, 20121 Milan, Italy; 3 Department of Clinical and Experimental Sciences, University of Brescia, 25121 Brescia, Italy; 4 NeMO-Brescia Clinical Center for Neuromuscular Diseases, 25064 Brescia, Italy; 5 Unit of Neurology, ASST Spedali Civili, 25123 Brescia, Italy; 6 Department of Molecular and Translational Medicine, University of Brescia, 25121 Brescia, Italy; 7 Neurology Unit Azienda Socio Sanitaria Territoriale Papa Giovanni XXIII, 24127 Bergamo, Italy; 8 Neuromuscular Omnicentre (NEMO), Fondazione Serena Onlus, 20162 Milan, Italy; 9 Laboratory of Neuroscience, Department of Neuroscience, IRCCS Istituto Auxologico Italiano, 20149 Milan, Italy; 10 Neuroncology/Neuroinflammation Unit, IRCCS Mondino Foundation, 27100 Pavia, Italy; 11 Neurology Unit, IRCCS Ospedale San Raffaele, 20132 Milan, Italy; 12 Neurology Unit, Manzoni Hospital, ASST Lecco, 23900 Lecco, Italy

**Keywords:** amyotrophic lateral sclerosis, survival, long-term survivors, prognostic, epidemiology, SLALOM

## Abstract

**Background**: Amyotrophic Lateral Sclerosis (ALS) is a fatal neurodegenerative disease with variable clinical progression. While median survival is 2–4 years, 5–15% of individuals survive for longer. **Methods**: We conducted a retrospective, observational study using a population-based ALS register in Lombardy, Italy, to identify the clinical characteristics of long-term ALS survivors (≥10 years). Incident cases included in two periods (1998–2002 and 2008–2012) were considered. **Results**: A total of 828 ALS cases were included. Median survival for the entire cohort was 2.2 years (IQR 1.1–4.4). However, long-term survival was observed in 7% of individuals at 10 years, and 3% at 15 years. Long-survivors had a median survival of 13.4 years, significantly longer than the 1.9 years of non-long-survivors (IQR 1.0–3.6). Long-survivors were younger at disease onset and diagnosis, had longer diagnostic delay, and were more likely to have had a spinal onset. The cohort also showed a higher proportion of males among long-term survivors (75% vs. 59%). No significant difference in survival was observed between the two examined periods. **Conclusions**: Our findings suggest that long-term ALS survival is likely influenced by a complex interplay of clinical, genetic, and environmental factors, along with the intrinsic rate of motor neuron degeneration.

## 1. Introduction

A reliable prognostication of clinical course and survival in amyotrophic lateral sclerosis (ALS) seems uncertain, yet the disease is invariably deemed fatal, with median survival of 2–4 years [1,2,3]. The variability in clinical progression is traditionally thought to be related to the phenotypic heterogeneity of ALS, this being regarded as a possible predictor of longer survival, for example, in ALS with predominant upper motor neuron involvement [4,5]. Nowadays the increasing resources provided by genetic analysis are disentangling to some extent the issue, because a growing number of genes are characteristically related to slow- or fast-progressing disease, both in familial and sporadic ALS [6,7]. On the other hand, epidemiologic data provided by population-based registries are consistent with an estimate of 5–15% long-surviving ALS subjects [8], with some clinical features being recognized as good predictors of long survival: for example, spinal onset, young age of onset, and a long diagnostic delay [9,10]. Of note, there is no full agreement on the definition of long survival: in some instances, this is more than 5 years [11]; in others, it is more than 10 years.

Our aim is to investigate the clinical features of individuals with ALS who survived for 10 years or more and who were enrolled in a large population-based register of ALS patients established in Lombardy, Italy. Two five-year periods were taken into consideration, 1998–2002 and 2008–2012, in order to explore and describe clinical courses and main predictors distinguishing long-survivors from the general ALS population, and to identify possible differences over time, if any.

## 2. Materials and Methods

### 2.1. Data Collection

SLALOM (SLA LOMbardia) is a population-based register of ALS patients resident in the Lombardy region of Italy, an area of about 10 million inhabitants. SLALOM was established at the Mario Negri Institute for Pharmacological Research IRCCS, Milan in 1997. Briefly, a widespread network of local neurologists and neurophysiologists enroll every subject identified as a definite, probable, probable laboratory-supported, or possible case of ALS according to the revised El Escorial criteria. Diagnoses are refined (confirmed, changed, or rejected) during follow-up through on-site visits and detailed reviews of clinical records. Suspected ALS cases are accepted only if clinical progression is verified; such cases are subsequently allocated into a higher degree of diagnostic certainty.

Survival was calculated from date of diagnosis until date of death or of last follow-up (30 June 2024). If known, date of death was recorded by local investigators. If date of death was missing, an inquiry was sent to the municipal registry office of residency. We defined long-survivors as those surviving 10 years or more. The whole process was managed according to the General Data Protection Regulation (EU) 2016/679 (GDPR), after approval by local ethical committees.

### 2.2. Statistical Methods

Descriptive statistics were obtained for the entire sample, and long-survivors were compared with non-long-survivors. Continuous variables were reported as median with interquartile range (IQR) or range, categorical variables as count and percentage. Comparisons of long-survivors with non-long-survivors were carried out with the Mann–Whitney–Wilcoxon test for continuous variables; for categorical variables, the chi-square test or Fisher’s exact test was used. Survival was described for the entire sample and separately for long-survivors and non-long-survivors with Kaplan–Meier survival curves. The significance level was set at 0.05, and tests were two-tailed. Analyses were performed with the SAS statistical package (version 9.4, SAS Institute, Cary, NC, USA).

## 3. Results

The total number of incident ALS cases included in the analyses was 828, with 401 recruited for the period 1998–2002 and 427 for 2008–2012. Data on demographics, clinical features at diagnosis, and survival periods were all fully available, whereas data on main clinical milestones (tube feeding, mechanical ventilation) were not available in some cases (Table 1). The proportion of females/males was 45%/55%. The median age of onset was 65 years (IQR 57–72), the median age at diagnosis was 66 years (IQR 58–73), and the median diagnostic delay was 9 months (IQR 5–13). Data on other clinical characteristics are presented in Table 1. Definite, probable, and possible ALS accounted for 84.7% of cases. All the 127 subjects categorized as suspected ALS category at diagnosis were followed; in these cases, diagnosis was confirmed during follow-up.

**Figure 1:**
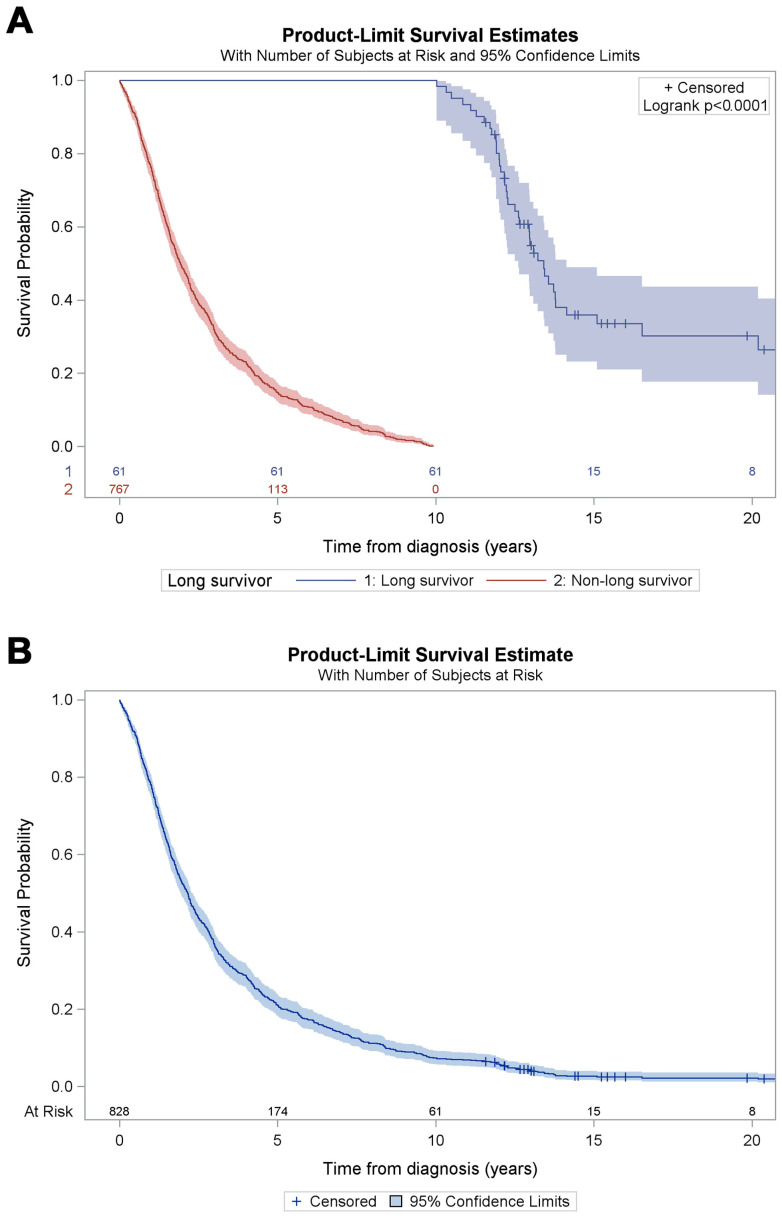
(**A**) Kaplan–Meier survival curves for non-long-survivors and long-survivors. (**B**) Kaplan–Meier survival curve for the entire sample.

The median estimated survival for the entire sample was 2.2 years (IQR 1.1–4.4). The cumulative survival probability was 21% at 5 years, 7% at 10 years, 3% at 15 years, and 2% at 20 years (Figure 1B).

The number of ALS subjects who survived 10 years or longer after diagnosis (long-survivors) was 61 (7%). No significant differences were identified between the two periods (1998–2002 vs. 2008–2012) (Table 1). The clinical features of the groups of individuals who survived <10 years and ≥10 years are shown in Table 1. Long-survivors were younger at both disease-onset and diagnosis, and their diagnostic delay was three months longer. Among long-survivors, 13 subjects were diagnosed as having suspected ALS according to El Escorial criteria; however, all of these individuals were subsequently categorized as having definite, possible, or probable ALS during follow-up. Long-survivors more frequently had had a spinal onset (75% vs. 59% in non-long-survivors), and a higher proportion of them were males (74% vs. 54% of non-long-survivors). Riluzole was started within one month of diagnosis in 75% of cases where data were available. The proportions of subjects with tube-feeding or non-invasive ventilation and of those treated with riluzole were not significantly different in long-survivors vs. non-long-survivors. Median survival in long-survivors was 13.4 years (12.0-not estimable), whereas it was 1.9 years (IQR 1.0–3.6) in non-long-survivors. Cumulative survival probability in long-survivors vs. non-long-survivors is shown in Figure 1A.

Among 61 long-survivors, information about tracheostomy was not available for 17 subjects. Among the other 44, tracheostomy was performed on 11 individuals, all of whom were males. The site of disease onset was spinal in nine subjects and bulbar in two. The median time of performing the procedure was 7.5 years after diagnosis. The main characteristics of the 11 long-survivors on whom tracheostomy was performed are described in Table 2. Information about genetic testing was available for 26 subjects (Table 3). Among these, 12 underwent genetic testing, and a TDP-43 mutation was identified in one subject.

## 4. Discussion

Prognosis of ALS and the rate of progression of the disease both encompass a noticeable variability. Population-based registers indicate that only a small proportion of ALS individuals, an estimated 5–15% of the general ALS population, are long-surviving [8,12]. Herein we describe the main clinical features of subjects who were enrolled in a population register of ALS and who survived 10 years or longer. Our study aim was to highlight predictors of long-term survival over time. Median survival in our sample was 2.2 years: 13.4 years in long-survivors and 1.9 years in non-long-survivors. The proportion of ALS long-survivors in the entire sample was 7%. Previous studies report approximately 5% to 10% of ALS patients surviving 8 years or more [6,8]. No significant difference was observed between the two examined periods, with proportions of long-survivors being, respectively, 7% and 8%. It is likely that the improvement in ALS prognosis observed in recent years [13] has not affected survival in long-survivors, but has had an effect on non-long-survivors. Improvement in the multidisciplinary supportive care provided by tertiary centers in recent years may explain this. Due to the lack of collected data on supportive care, any inference regarding its beneficial impact on patient survival remains highly speculative. In partial agreement with our own previous observations [3] and the findings of other studies [12,14,15], we found four predictors of long-term survival: lower age at onset, longer diagnostic delay, male gender, and spinal onset (see Supplementary Table S1). The median age of onset was significantly lower in long-survivors, in line with previous studies which assumed old age as a risk factor for shorter survival [9,12,14]. In our study, the diagnostic delay in long-survivors was 12 months, significantly longer than that of non-long-survivors (9 months). Males and subjects with spinal onset were more prevalent among long-survivors, probably due to overrepresentation of bulbar onset, which is associated with shorter survival [9,10,12,14] in females.

Fewer than half of subjects alive after 10 years underwent measures possibly affecting survival such as percutaneous gastrostomy, non-invasive ventilation, or tracheostomy. With regard to the remaining cases, the possibility of a slow progression of the disease must be taken into account.

A correlation between the use of riluzole and survival was not evaluated because these data were only available for 51% of sample subjects. In addition, 75% of subjects started treatment with riluzole within 1 month of the date of ALS diagnosis, and only a minority of cases reported having stopped the treatment during follow-up. In consequence, it was not feasible to evaluate whether riluzole prolonged survival in the last clinical stage of ALS [16]. The possibility of misdiagnosis cannot be completely excluded; however, all our subjects were followed by expert neurologists, and we included in this survey only suspected ALS cases who had a subsequent re-evaluation congruous with definite, possible, or probable ALS.

This study has some strengths. First of all, as a population-based study, it could provide a real-world picture of clinical course and survival of the disease. Moreover, follow-up was prolonged, with repeated visits by neurologists and thorough investigations to re-assess diagnoses that were confirmed across the entire sample. Collecting all incident ALS cases in two periods, a decade apart, could have allowed for the detection of differences in survival over time.

Nevertheless, our survey has some limitations. Unfortunately, the dates of the main clinical milestones (tube feeding, mechanical ventilation) for examined ALS individuals were not available in some cases, so these could not be related to clinical progression and survival. Most importantly, the presence/absence of tracheostomy was unknown in a proportion of long-survivors; for this reason, it was not possible to evaluate tracheostomy-free survival. Genetic testing was performed in only a very small proportion of subjects, probably only those who were admitted to ALS referral centers; consequently, no association with survival could be evaluated.

In conclusion, it is likely that long-term ALS survival results from a complex interplay of clinical factors, genetic variations, and the intrinsic rate of motor neuron degeneration. The observation of cases exhibiting prolonged survival independent of respiratory and nutritional support suggests the existence of unknown genetic and environmental modifiers. Targeted research into such long-survivors offers a promising avenue for unravelling the biological basis of slow ALS progression. In addition, a more reliable prognostication of ALS could favorably influence ALS care and economic resource allocation.

## Figures and Tables

**Table 1:** Descriptive statistics for the entire sample and for long-survivors vs. non-long-survivors.

	Total (n = 828)		Survival < 10 Years (n = 767)		Survival ≥ 10 Years (n = 61)		*p*-Value
	Median	IQR	Median	IQR	Median	IQR	
**Follow-up duration**	2.2	1.1–4.4	1.9	1.0–3.6	12.9	12.0–14.5	<0.0001
**Age at diagnosis**	66.3	58.2–73.0	67.2	59.1–73.4	57.2	48.2–63.8	<0.0001
**Age at onset**	65.4	57.1–72.2	66	58.0–72.6	55.5	45.2–62.4	<0.0001
Diagnostic delay * (months)	9.1	5.5–13.4	9	5.4–13.1	12.1	8.0–21.1	0.0003
	**n**	**%**	**n**	**%**	**n**	**%**	
**Diagnosis period**							0.4986
1998–2002	401	48.4	374	48.8	27	44.3	
2008–2012	427	51.6	393	51.2	34	55.7	
**Sex**							0.0024
Female	371	44.8	355	46.3	16	26.2	
Male	457	55.2	412	53.7	45	73.8	
**El Escorial category at diagnosis**							0.0555
Definite ALS	311	37.6	297	38.7	14	23	
Probable ALS	303	36.6	279	36.4	24	39.3	
Possible ALS	87	10.5	77	10	10	16.4	
Suspected ALS	127	15.3	114	14.9	13	21.3	
**Site of onset**							0.0108
Bulbar/generalized	331	40	316	41.2	15	24.6	
Spinal	497	60	451	58.8	46	75.4	
**PEG**							0.8174
No	318	62	285	61.8	33	63.5	
Yes	195	38	176	38.2	19	36.5	
Missing	315		306		9		
**NIV**							0.2361
No	308	60.6	281	61.5	27	52.9	
Yes	200	39.4	176	38.5	24	47.1	
Missing	320		310		10		
**Riluzole**							0.0916
No	72	17.1	62	16.1	10	27	
Yes	350	82.9	323	83.9	27	73	
Missing	406		382		24		

* Time from onset to diagnosis in months.

**Table 2:** Characteristics of long-survivors subjected to tracheostomy.

Subject	Year of Onset	Sex	Site of Onset	Year of Diagnosis	Status	Survival Time from Diagnosis (Years)	Tracheostomy-Free Survival Time (Years)
1	1999	Male	Spinal	2000	Dead	13.8	12.2
2	2001	Male	Spinal	2001	Alive	22.4	12.7
3	2000	Male	Spinal	2001	Alive	22.6	8.2
4	2008	Male	Bulbar	2009	Dead	11.9	0.9
5	2011	Male	Spinal	2012	Dead	12.3	5.1
6	2009	Male	Spinal	2009	Alive	14.5	8.2
7	2010	Male	Spinal	2011	Dead	13.4	3.3
8	2009	Male	Spinal	2009	Dead	12.9	2.7
9	2006	Male	Bulbar	2008	Alive	14.4	12.7
10	2008	Male	Spinal	2009	Dead	13.7	6.9
11	2006	Male	Spinal	2008	Alive	15.4	Not available

**Table 3:** Characteristics of long-survivor subjects and gene evaluations.

Subject	Gender	Year of Onset	Site of Onset	Year of Diagnosis	Status	Genetic Test Performed	Mutation Identified
A	Male	2009	Bulbar	2010	Dead	None	
B	Male	2008	Spinal	2010	Dead	None	
C	Female	1999	Spinal	2001	Dead	None	
D	Female	2009	Spinal	2011	Dead	None	
E	Female	2001	Spinal	2002	Alive	None	
F	Female	2000	Spinal	2004	Alive	None	
G	Male	2011	Spinal	2012	Dead	None	
H	Male	2010	Spinal	2011	Dead	None	
J	Male	2008	Spinal	2009	Dead	None	
K	Male	2004	Spinal	2009	Alive	None	
I	Female	2012	Spinal	2012	Alive	None	
L	Male	2008	Spinal	2009	Dead	None	
M	Male	2008	Spinal	2009	Dead	None	
N	Male	2009	Spinal	2009	Dead	None	
O	Male	2008	Bulbar	2009	Dead	SOD1, FUS, TDP43, C9ORF72	None
P	Male	2011	Spinal	2012	Dead	SOD1, FUS, TDP43, C9ORF72	None
Q	Male	2010	Spinal	2011	Dead	SOD1, FUS, TDP43, C9ORF72	None
R	Female	2010	Spinal	2010	Dead	SOD1, FUS, TDP43, C9ORF72	TDP43
S	Female	2010	Bulbar	2011	Dead	SOD1, FUS, TDP43, C9ORF72	None
T	Female	2010	Spinal	2011	Alive	SOD1, FUS, TDP43, C9ORF72	None
U	Male	2011	Spinal	2012	Alive	SOD1, FUS, TDP43, C9ORF72	None
V	Female	2011	Spinal	2011	Alive	SOD1, FUS, TDP43, C9ORF72	None
W	Male	2006	Spinal	2008	Alive	SOD1, FUS, TDP43, C9ORF72	None
X	Female	2007	Bulbar	2008	Alive	C9ORF72	None
Y	Male	2008	Spinal	2009	Dead	SOD1	None
Z	Male	2011	Spinal	2012	Alive	SOD1, FUS, TDP43, C9ORF72	None

## Data Availability

Anonymized data are available from the corresponding author upon request. The dataset generated in this study is available in the Zenodo repository https://zenodo.org/communities/irfmn-irccs?q=&l=list&p=1&s=10&sort=newest (it will be available from 13 October 2025).

## Supplementary Materials

The following supporting information can be downloaded at: https://www.mdpi.com/article/10.3390/jcm14207351/s1, Table S1: Comparing predictors of long survival among studies. References [3,12,14,15] are cited in the Supplementary Materials.
