# Supplementary material for: Understanding Long-Term Survival in ALS: A Cohort Study on Subject Characteristics and Prognostic Factors
Source: J Clin Med. 2025 Oct 17;14(20):7351. doi: 10.3390/jcm14207351 (PMC12565721; doi:10.3390/jcm14207351)
Supplement: Supplementary file 1 [file jcm-14-07351-s001.zip › jcm-3880743-supplementary.pdf]

Suplementary Table S1. Comparing predictors of long survival among studies.

	N° of subjects	Survival rate	Predictors of long survival								
			Male sex	Spinal onset	Younger age at diagnosis	Longer diagnostic delay	Lower rate of change before diagnosis	Higher FVC at baseline	Predominant upper motor signs	Rate of disease progression	El Escorial category at diagnosis
Present study	828	7%>10ys	yes	yes	yes	yes	Not evaluated	Not evaluated	Not evaluated	Not evaluated	no
[3] Bianchi E, Pupillo E, De Feudis A, Enia G, Vitelli E, Beghi E. Trends in survival of ALS from a population-based registry. *Amyotroph Lateral Scler Frontotemporal Degener*. 2022 Aug;23(5-6):344-352.	502	9%>10ys	yes	yes	yes	yes	Not evaluated	Not evaluated	Not evaluated	Not evaluated	Not evaluated
[12] Chiò A, Logroscino G, Hardiman O, et al. Prognostic factors in ALS: A critical review. *Amyotroph Lateral Scler.* 2009 Oct-Dec;10(5-6):310-23.	Sistematic review	5-10%>10ys	no	yes	yes	yes	Not evaluated	yes	Not evaluated	yes	yes
[14] Czaplinski A, Yen AA, Appel SH. Amyotrophic lateral sclerosis: early predictors of prolonged survival. *J Neurol*. 2006 Nov;253(11):1428-36.	1034	28%>5ys	no	no	yes	yes	yes	yes	Not evaluated	yes	Not evaluated
[15] Zoccolella S, Beghi E, Palagano G, et al. Predictors of long survival in amyotrophic lateral sclerosis: a population-based study. *J Neurol Sci.* 2008 May 15;268(1-2):28-32.	128	10%>8ys	no	no	yes	yes	Not evaluated	Not evaluated	yes	Not evaluated	no
